# Multicomponent Reactions Accelerated by Aqueous Micelles

**DOI:** 10.3389/fchem.2018.00502

**Published:** 2018-10-22

**Authors:** Daniel Paprocki, Arleta Madej, Dominik Koszelewski, Anna Brodzka, Ryszard Ostaszewski

**Affiliations:** Institute of Organic Chemistry, Polish Academy of Sciences, Warsaw, Poland

**Keywords:** multicomponent reactions, water, micelles, surfactants, on water reaction

## Abstract

Multicomponent reactions are powerful synthetic tools for the efficient creation of complex organic molecules in an one-pot one-step fashion. Moreover, the amount of solvents and energy needed for separation and purification of intermediates is significantly reduced what is beneficial from the green chemistry issues point of view. This review highlights the development of multicomponent reactions conducted using aqueous micelles systems during the last two decades.

## Introduction

One of the major principles of green chemistry is the development of environmentally friendly chemical protocols and technologies. On the basis of this fact, the selection of an environmentally benign solvent has gained much attention. One of the most green medium is water as it is cheap and readily available, what has become a major concern in organic synthesis. Moreover, the use of water as a solvent for organic transformations has received considerable attention since it is an inexpensive, non-toxic and non-flammable medium (Li, 2005; Dallinger and Kappe, 2007; Chanda and Fokin, 2009). In addition, reactions conducted in aqueous media reveal unique reactivity and selectivity that are not usually detected in organic solvents (Cornils, 1995; Kobayashi et al., 1999). However, many organic transformations in aqueous media are limited in scope due to poor solubility of the reactants. To overcome this limitation the polar aprotic reaction media were changed to an “on water” approach, involving also surfactants that allow a micelle-promoted reaction to be run in aqueous medium. Nevertheless, a number of heterocyclic compounds including acridines (Wang et al., 2005), furans (Wnorowski and Yaylayan, 2000), indoles (Cho et al., 2000), pyrazines (Totlani and Peterson, 2005), pyridines (Khadilkar et al., 1995), pyrimidines (Bose et al., 2003), and pyrazolines (Wang et al., 2007) have been successfully synthesized in aqueous media.

The use of detergents in concentration exceeding critical micelle concentration (CMC) is one of the most convenient methods to promote the reaction in aqueous medium. The presence of surfactants and detergents definitely improves the solubility of hydrophobic compounds in water. In several cases the rate of particular reaction was substantially enhanced (Dwars et al., 2005) what can be explained by at least three phenomenons. The first is associated with the increased substrates concentration in water in the area of the aggregates formed by the surfactants (e.g., micelles, vesicles). Second phenomenon is associated with a different polarity of the actual locus where the reaction proceeds. The last phenomenon is associated with micelles and vesicles structure what introduces a plausible steric hindrance and decreases the extent of side reactions (Walde et al., 2014). Therefore, as it was recently pointed out by Sorrenti et al. (2013), micellar systems behave much more as nano-reactors characterized by unique features than as a soapy version of homogeneous catalysis. Early review papers concerning the micellar catalysis date back to the late 1970's and describe catalysis directly performed by the supramolecular aggregates (Fendler and Fendler, 1970). From a certain point of view micelles can be compared with enzymes. Enzyme action is based on isolating substrates from the bulk solvent, improving its solubilization in water, favoring compartmentalization. As a result of compartmentalization the enhancement of substrates local concentration and reactivity, imparting unique regio- and stereoselectivities is observed (Sorella et al., 2015). Micelles are formed by amphiphiles in water or media similar to water are especially simple spherical supramolecules (Gratzel, 1991). Moreover, various types of aggregates in aqueous surfactant solutions were recognized like: vesicles, bilayers etc., however in this review we do not concentrate on morphology of these aggregates. The presence of micelles in reaction mixtures enables to accelerate or inhibit a given chemical reaction relative to the equivalent reaction in a pure aqueous medium (Morawetz, 1969; Fendler, 1975). In general, the influence of the presence of aggregates on the reaction course is known as “micellar catalysis.” Usually it refers to the acceleration of the reaction rate (Scrimin et al., 1998). Morawetz recognized and discussed three typical possibilities for micellar assistance on the reaction course. First, an amphiphilic reagent forms a micelle. The structure of a micelle is flexible and changeable during the reaction. Second possibility is associated with the interactions between the micellar surfactants what definitely may influence the reaction rate. The last possibility for micellar assistance is associated with the structure of surfactant catalytically active groups. It promote a formation of micelle possessing catalytically active groups and functions as a catalyst.

Additionally, the application of an aqueous micellar solution as a reaction medium improves overall process by simplifying product isolation and catalyst recycling. It is only possible when, during removal of the product, the catalyst is confined in the micellar aggregates. In other cases a different scenario influenced by the catalyst-surfactant interactions is possible. In the best-case scenario, the product can be isolated by simple extraction, leaving the catalyst and the surfactant in the micellar medium. Sometimes product purification is even simpler–product fall out from reaction mixture and can be filtrated or centrifuged from the reaction mixture, then reaction medium with micelles and catalyst can be reused for next reaction. Regarding to environmental issues which have likely been under consideration for some time, Lipshutz et al. evaluated the commonly performed reactions on the basis of their environmental (E) factors (Sheldon, 2007, 2008), one of the measures which illustrates the greenness of the reaction. In most cases, using aqueous micellar solution as a reaction medium reduces E factors by more than an order of magnitude (Lipshutz et al., 2013; Klumphu and Lipshutz, 2014).

Recently, a vast number of organic compounds were obtained using diverse synthetic transformations in water-surfactant systems, e.g., the thiolysis of *p*-nitrophenyl acetate, the decarboxylation of 6-nitrobenzisoxazole-3-carboxylate (Perez-Juste et al., 2000), an aza-Diels–Alder reaction (Costantino et al., 2009), or an *O*-sulfonylation/Knoevenagel condensation/hetero-Diels–Alder reaction cascade (Ghandi et al., 2013). Furthermore, Bruce Lipshutz's group developed a great number of various metal catalyzed reactions in the presence of surfactant aggregates (Bhattacharjya et al., 2015). Recently various transition metal–catalyzed reactions proceeded in water were revived by Lipshutz et al. (2018). Also Handa and co-workers have developed surfactant FI-750-M, which can successfully replace polar aprotic solvents like dioxane, *N*-methylpyrrolidone or DMF. FI-750-M was used for proceeding various Pd-catalyzed couplings, sulfonylation of polyfluoroarenes or oxyhalogenation of alkynes in an aqueous solution (Bihani et al., 2018; Finck et al., 2018; Smith et al., 2018). The surfactant structure which contains liphophilic region, the proline linker and hydrophilic mPEG region provides nanomicelles with different binding sites. Such arrangement could bring together substrates and catalyst, what together with hydrophobic effect, can lead to clean and fast product formation.

Multicomponent reactions (MCRs) have gained more attention in modern organic synthesis as the number of applications which they are notified increase significantly in medicinal chemistry, drug discovery programs, combinational chemistry, natural product synthesis, argochemistry and polymer chemistry (Simon and Li, 2012). MCRs are one-pot processes where three, four or more components are bringing together. Such protocols are of great interest in diversity-oriented synthesis, especially to generate compound libraries for screening purposes. MCRs have been commonly applied in heterocyclic and combinatorial chemistry (Dömling et al., 2012; Graaff et al., 2012; Brauch et al., 2013). In addition, MCRs conducted in water as a reaction medium are one of the most powerful approach toward the atom efficient synthesis of bioactive molecules with minimal added cost or waste (Pirrung and Sarma, 2004; Chanda and Fokin, 2009). On the basis of these facts, the using of water as a green solvent for organic multicomponent reactions is of great interest to many research groups as it constitutes one of the highlighted subclasses of ideal synthesis.

**Figure 1:**
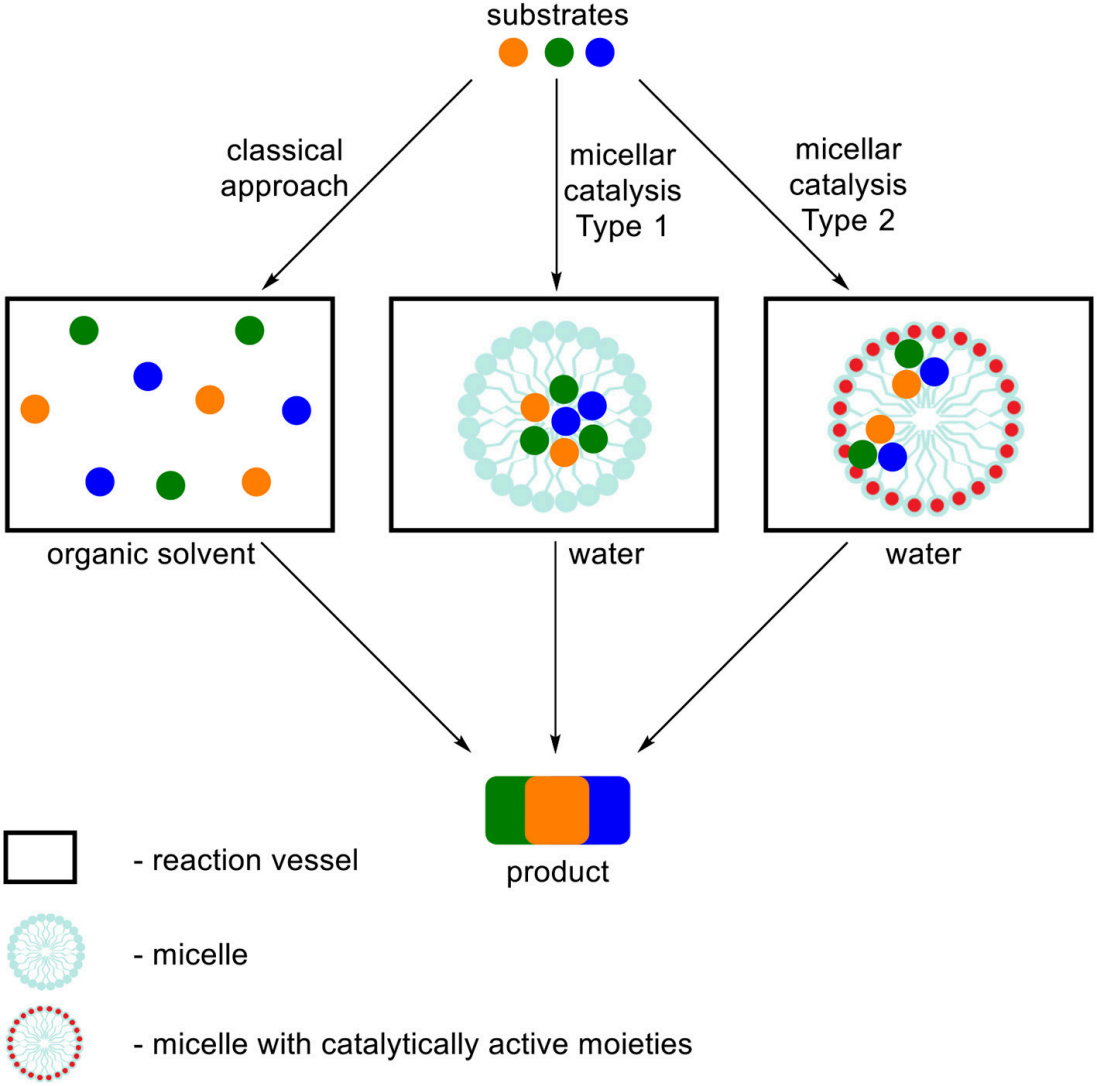
Schematic representation of two types of micellar catalysis summarized in this review in respect to classical approach for MCRs.

In this review we focused on the literature describing MCRs performed in aqueous surfactant systems, in respect to classical methods. In order to organize the influence of the type of used surfactant on the MCRs we divided the term “micellar catalysis” into two types (Figure 1). First one refers to aggregates (we do not distinguish its morphology) increasing solubilization of substrates, creation of local environment beneficial for a reaction course and enhancement of local concentration and reactivity by simplifying the meeting of three or more substrates at the same time in the volume of aggregate. A variety of surfactants are commonly used for this type of catalysis, which do not possess catalytically active moieties. Under the classical approach, when all substrates are dissolved in homogenous medium (or some of them are precipitated), reactants are chaotically dispersed in a whole volume what substantially affects their local concentration. The second type of micellar catalysis not only influences the reaction by the factors defined for the first type, but also possessing catalytically active functional groups in the surfactant structure, which act as a conventional catalyst. This approach significantly simplifies the meeting of substrates with the catalytically active moieties, which are the part of aggregate. The most often used surfactants in this type of catalysis are dodecylbenzenesulfonic acid (DBSA) and various Lewis acid-surfactant-combined catalysts (LASCs), which are usually salts of transition metal and dodecyl sulfate or the other lipophilic acid.

## The mannich reaction

The Mannich reaction is a multicomponent reaction first described at the beginning of twentieth century (Mannich and Krosche, 1912). This three-component reaction employs amine, enolizable ketone and non-enolizable aldehyde providing β-aminoketone. The reaction is atom efficient, as the only side product is a molecule of water. The Mannich reaction products are often building blocks of valuable compounds (Arend et al., 1998). Asymmetric variants of the Mannich reaction often utilize proline and its derivatives as catalysts (List, 2000; List et al., 2002; Marques, 2006). Mannich reaction can be performed in an aqueous solution, however an efficient reaction course requires acid, Lewis acid or base catalytic system which needs to be compatible with water and micellar aggregates (micellar catalysis type 1), or surfactant which possesses catalytically active moieties (micellar catalysis type 2).

Systems exhibiting micellar catalysis type 1 were described by Lu and Cai (2010). They have developed catalytic system comprising 5% Triton X-10 solution and perchloric acid which efficiently catalyzed the Mannich reaction at room temperature. Without the surfactant addition the yield of a model reaction was poor (19 vs. 92% in the presence of Triton X-10), so it efficiently promoted reaction, without the acid catalyst only a trace of product was obtained. Mannich reaction under such conditions was reported to be faster than performed in the presence of homogenous catalyst like H_2_SO_4_ and allowed for reaching yields 83–98% after 6 h at room temperature for 20 different derivatives. Additionally, the separation of product was very straightforward, the precipitated product was filtered off, washed with water and dried. Akiyama et al. (2005) have described the catalytic system including 10 mol% of SDS and HCl, for stereoselective catalysis reaction between aromatic aldehydes, anilines and cyclohexanone. For 12 examples of products *anti:syn* isomers ratios were between 77:33 and 99:1, with high yield between 83 and 90%. For the model reaction the yield and selectivity were higher than in the other catalytic systems including reactions performed in organic solvents such as acetonitrile or methanol. Kumar et al. (2011) described Mannich reaction of 4-hydroxycoumarin, formaldehyde and secondary amines, performed in 100 mM Triton X-100 solution. 14 benzylaminocoumarin derivatives were synthetized according to a very simple procedure: substrates were stirred at room temperature in aqueous surfactant solution and, after few hours, the precipitated products were filtered off and recrystallized from ethanol. Sahu et al. (2014) developed the protocol for the efficient synthesis of 2-aminobenzothiazolomethylnaphthols and 5-(2-aminobenzothiazolomethyl)-6-hydroxyquinolines starting from various aromatic aldehydes, 2-aminobenzothiazole and 2-naphthol or 6-hydroxyquinoline. In this protocol substrates were stirred in water at room temperature. The reaction did not occur in organic solvents, and only traces of product were obtained under neat conditions. On the other hand, an aqueous solution of SDS provided high yields (up to 96%). Moreover, this catalytic system can be reused four times without significant loss of yield, after filtering off participated products. Kumar et al. (2010b) showed that the same reaction can be also performed at higher temperature up to 100°C. Another work of this author (Kumar et al., 2010a) dealt with the other variant of it–a micelle-promoted Mannich reaction for the synthesis of Betti bases starting from secondary amine, substituted benzaldehydes and β-naphthol. From several tested catalytic systems, Triton X-100 was determined to promote the most results of the reaction. The authors suggested the role of the surfactant in stabilizing and promoting dehydrative imine formation which is a characteristic feature of colloidal dispersion system. Also the usability of newly synthetized quaternary ammonium salt (QAS) gemini fluorosurfactants for catalysis of the Mannich reaction was investigated by Shen et al. (2008). One of surfactants synthetized from perfluorooctanesulfonyl fluoride, *N*,*N*-dimethylpropyl-1,3-diamine followed by quaternarization reaction with 4,4′-bis(chloromethyl)-1,1′-biphenyl, catalyzed the Mannich reaction under both acid and base conditions, outperforming in terms of yield other synthetized surfactants and some of the standard catalytic systems. The usability of fluorinated surfactant was confirmed on 15 various compounds synthetized with high yield up to 91%, however the stereoselectivity of products (if applicable) was very poor.

Another approach to the Mannich reaction in water is based on the use surfactant that is also an acid catalyst (micellar catalysis type 2). This protocol allows to reduce the amount of additives to the reaction, what is desirable from the green chemistry point of view. The most common surfactant-catalyst for this reaction is dodecylbenzenesulfonic acid (DBSA)—its catalytic usability for the Mannich reaction was proved by numerous research groups. Manabe and Kobayashi (1999) showed that DBSA can be successfully applied for catalysis of the reaction between benzaldehyde, aniline and acetophenone resulting in higher yields comparing to surfactant/acid catalytic system such as SDS/TsOH or Lewis acid-surfactant catalyst e.g., Sc(O_3_SOC_12_H_25_)_2_. Moreover, no reaction occurred in organic solvents such as DCM or MeOH. The authors proved the usability of DBSA for 12 examples of Mannich reactions, as various products have been synthetized with almost quantitative yields, after 12 h at 23°C. Filho et al. (2015) have described DBSA-catalyzed Mannich reactions from aminonaphtoquinones. DBSA was determined to be more efficient catalyst than SDS, SDS/TsOH or Tween 80, proving that surfactant–catalyst can be successfully applied for the Mannich reactions. Also Chang et al. (2014) have prepared a series of Brønsted acid-surfactant-combined catalysts, SO_3_H-fuctionalized ionic liquids with different acids and tested their usability for the Mannich reaction. Among obtained catalysts, 3-(*N*,*N*-dimethyloctylammonium) propanesulfonic acid toluene sulfate ([DOPA][Tos]) provided the best catalytic activity due to the formation of emulsions during the reaction proceeded for 6 h at 25°C. The catalyst can be recycled nine times. Simple separating process allowed to repeat the reaction without any decrease of catalyst activity. The usability of catalytic system was confirmed on 10 various derivatives synthetized with high yield-up to 89%.

**Scheme 1:**
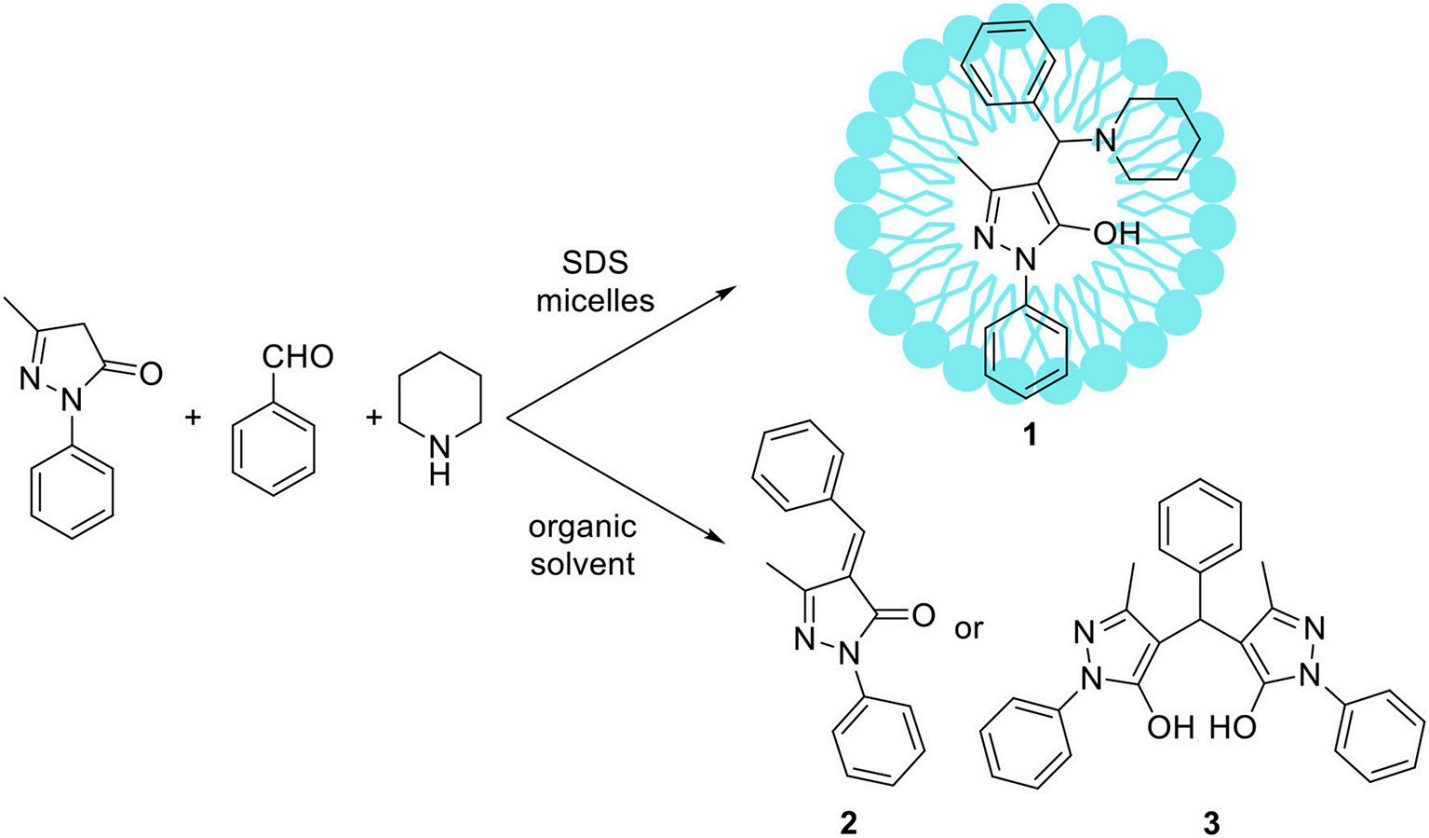
Comparison of Mannich-type reaction proceeded in aqueous micellar solution vs. organic solvent.

**Scheme 2:**
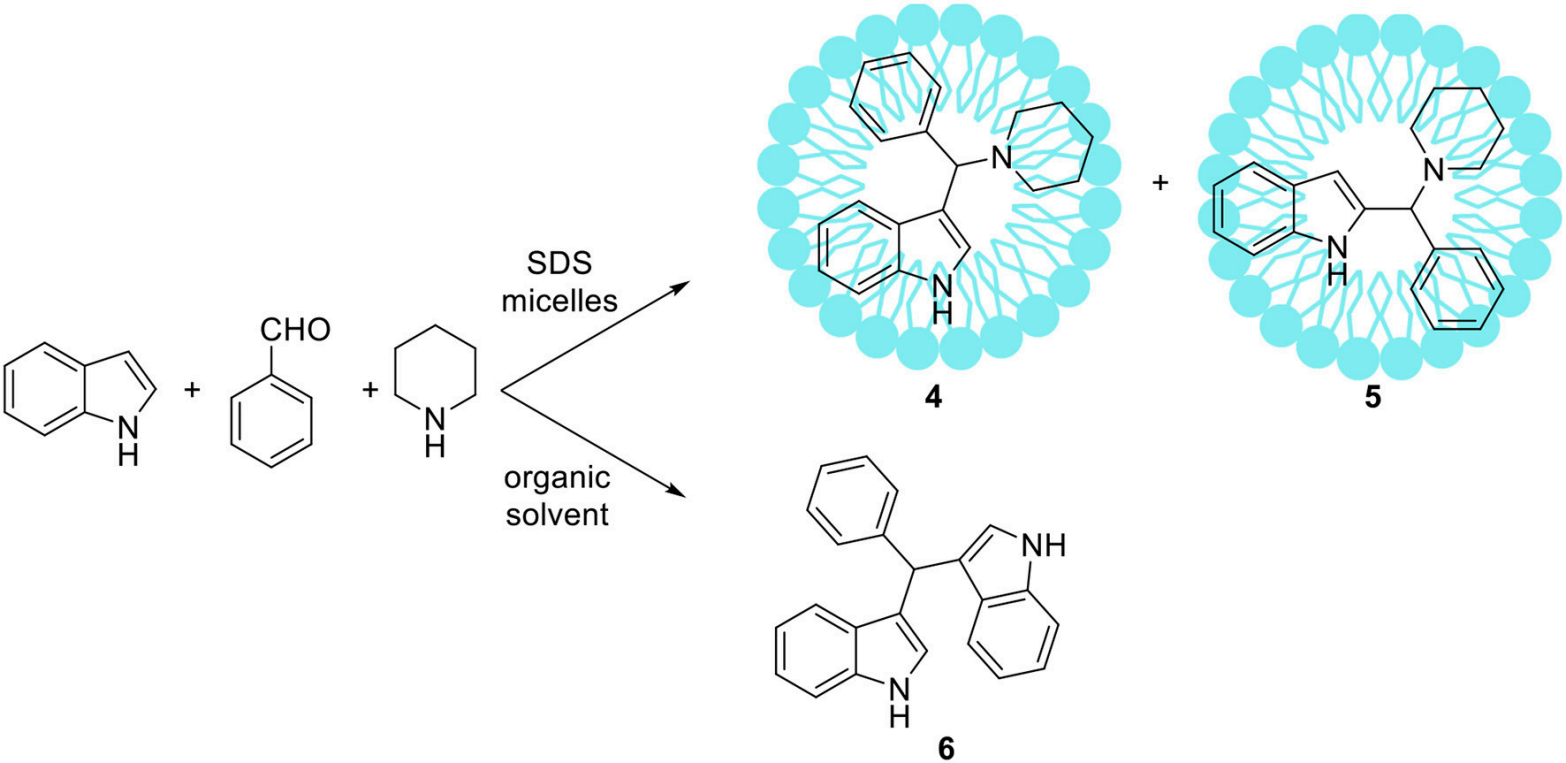
An unusual Mannich type reaction of indole reported by Kumar et al. (2013).

Some atypical variants of Mannich-type reactions were proceeded in aqueous surfactants systems. For example, Kumar et al. (2014) reported the reaction between a 3-methyl-1-phenyl-5-pyrazoline, an aldehyde and a secondary amine, followed by product aromatization to 4-amino alkylated 1-*H*-pyrazol-5-ol derivatives (**1**) (Scheme 1). The reaction occurred poorly in organic solvents; mostly the Knoevenagel type product **2** or dimers **3** were obtained (Scheme 1). However the reaction conducted in water/SDS system for 2 h at 80°C resulted in the desired products with almost quantitative yields. Another variant of the Mannich reaction, employing silyl enols instead of ketone, was described by Akiyama et al. (2002) and Manabe et al. (2001). The reactions were performed in water-organic solvent or water-surfactant systems at room temperature in the presence of catalytic amount of HBF_4_ or DBSA, which can be both surfactant and catalyst. Both catalytic systems can be successfully applied for this reaction with moderate to high yields. Also carbamates can be used instead of amine for the Mannich-type reactions, for example Yang et al. (2013) reported the reaction between naphthol, aromatic aldehyde and carbamates, which occurred in water/Tween 20 system at high temperature (75–80°C). The authors have synthetized 18 various product derivatives with almost quantitative yields, comparable to reactions performed in the presence of various acid or Lewis acid catalysts. The catalytic system was reused up to six times without significant loss of reaction yield. Kumar et al. (2012) reported the Mannich reactions from urea and thiourea in the presence of SDS/Cellulose-SO_3_H catalytic system. The authors carefully optimized reaction conditions including various acid and Lewis acid catalysts, and surfactants which allowed to perform reactions on 28 various substrates with high yield. The catalytic system can be also reused up to four times without significant loss of reaction yield. Kumar et al. (2013) developed unusual Mannich type reaction between indole, aromatic aldehyde and secondary amine providing 3-amino alkylated indoles with high selectivity (Scheme 2). In the presence of an organic solvent and various catalytic systems, the mixture of products **4**, **5**, and **6** was obtained, while in water-SDS system the desired product **4** was obtained with high selectivity. Under optimized conditions 25 derivatives of 3-amino alkylated indole were synthetized with 78–94% yields.

**Scheme 3:**
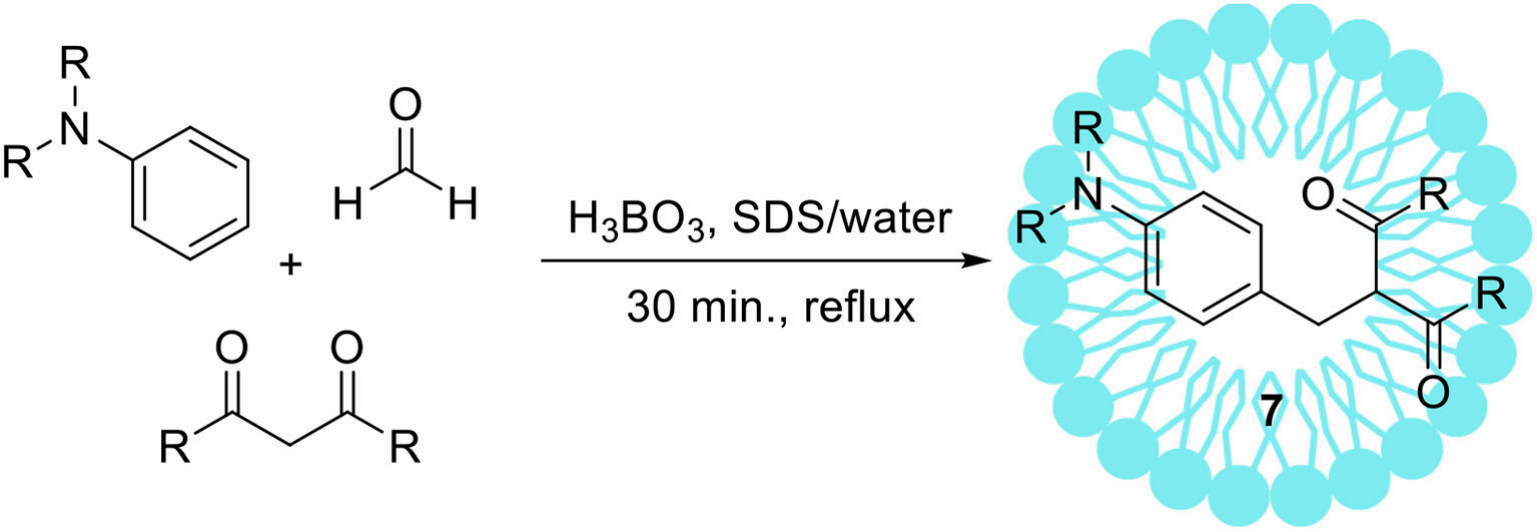
Mannich-type reaction with tertiary aromatic amines.

Kumar and Maurya (2008a) reported another example of an unusual Mannich type reaction using tertiary aromatic amines as substrates (Scheme 3). The authors optimized reaction conditions+ obtaining products of the structure **7** with the highest selectivity and yield. Boric acid and SDS turned out to be the best of tested catalytic systems. Afterwards, the authors synthetized 14 various products from different aromatic amines and 1,3-dicarbonyl compounds with high yield, up to 86%.

## The biginelli reaction

The Biginelli reaction, described in 1893 enables the obtaining of 3,4-dihydropyrimidin-2-(1*H*)-ones from an aldehyde, β-ketoester and urea. These moieties are very common in a variety of biologically active compounds, including natural products and medicines (Kappe, 2000; Blasco et al., 2010; Kaan et al., 2010), making the Biginelli reaction one of the most powerful and valuable multicomponent transformation. Unfortunately, originally this reaction was performed in organic solvents under quite harsh acidic conditions (Salehi et al., 2003; Chen et al., 2007). In the last few years a number of protocols have been established to overcome this drawback (Shen et al., 2010; Tamaddon et al., 2010; Saha and Moorthy, 2011). However, most of them involve expensive and toxic reactants, the stoichiometric amount of catalyst resulting in unsatisfactory yields or generation of byproduct which hampers the isolation of products.

**Scheme 4:**
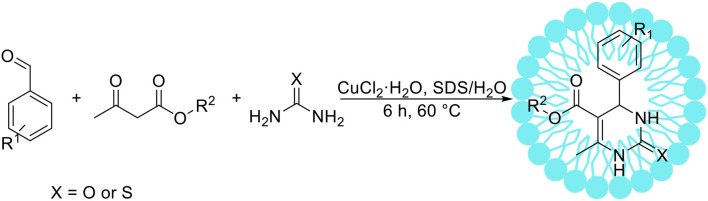
The Biginelli reaction.

Several research groups have reported the Biginelli reaction in an aqueous solution using protonic or Lewis acids as catalyst (Suzuki et al., 2006; Kalita and Phunkan, 2007), however these catalysts are often harmful, expensive and unstable. Xu et al. (2008) have developed environmentally benign approach for the Biginelli reaction catalyzed by Lewis acid in an aqueous surfactant system (Scheme 4). After detailed optimization studies the authors have chosen CuCl_2_·2H_2_O as the most suitable catalyst and SDS as a surfactant. The beneficial influence of a surfactant was proved by the reactions between benzaldehyde, ethyl acetoacetate and urea, which resulted in 93% yield of the appropriate product in the presence of 20 mol% surfactant. Without SDS only traces of desired product were obtained. The authors applied the optimized reaction conditions for the synthesis of 17 new 3,4-dihydropyrimidin-2(1*H*)-one derivatives with high yield, up to 96%. Bigdeli et al. (2011) developed micellar catalysis type 2 system involving *p*-dodecylbenzenesulfonic acid, which acted as an acidic catalyst and surfactant (micellar catalysis type 2). This approach is especially beneficial from the point of view of green chemistry, as it allows for significant reduction of non-renewable additives required for catalysis. The authors showed that the Biginelli reaction with various substrates can be proceeded in water with high yields, resulting in 3,4-dihydropyrimidin-2-(1*H*)-one derivatives.

## Isocyanide-based multicomponent reactions

**Scheme 5:**
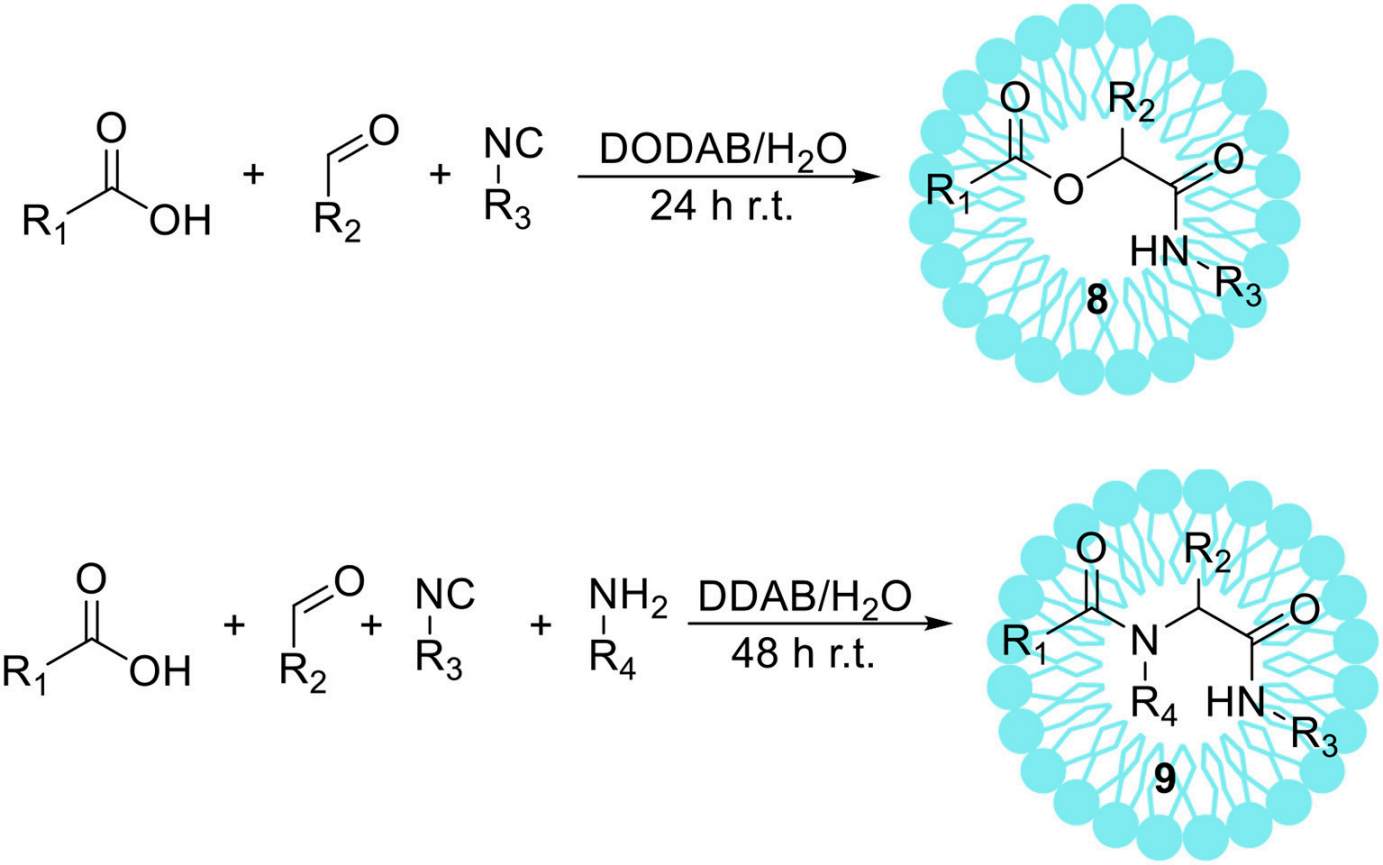
The Passerni (above) and the Ugi (below) reactions.

Isocyanide-based multicomponent reactions, such as a Passerini (P-MCR) and an Ugi (U-MCR) reaction, enable easy, and one-pot synthesis of peptidomimetics **8** and **9** from simple substrates (Scheme 5). These reactions have considerable potential in the creation of molecules being applied in combinatorial chemistry (Domling and Ugi, 2000; Boger et al., 2003; Domling, 2006). For example, U-MCR was used by Merck as a route to the anti-HIV drug Crixivan (Rossen et al., 1998). Even though these reactions are usually conducted in organic solvents (Neyer et al., 1990; Qu et al., 2011), there are several reports on using aqueous media as a solvent (Pirrung and Sarma, 2004; Vessally et al., 2011; Taran et al., 2014). We have shown that the addition of dioctadecyldimethylammonium bromide (DODAB), a cationic vesicle forming surfactant, noticeably improves the course of the Passerini reaction in aqueous solution leading to corresponding α-acyloxycarboxamides **8** with higher yields than under standard conditions in dichloromethane or water (Paprocki et al., 2015). After detailed studies on the reaction conditions 16 α-acyloxycarboxamide derivatives **8** were obtained with yields comparable to the same reactions proceeded in DCM. The reusability of the developed vesicle system was also investigated, showing that DODAB vesicle solution can be reused at least three times without significant decrease in yield. Additionally, we have shown that multicomponent reactions in micellar solution can be combined with enzymatic oxidation of alcohols in an aqueous surfactant medium (Paprocki et al., 2016). This protocol comprised the laccase from *Trametes versicolor*/TEMPO aerobic oxidation of a primary alcohol to aldehyde, followed by the Passerini reaction. However, these studies required careful optimization of reaction conditions including type and amount of surfactant, mediator, and pH-value to achieve maximum enzyme activity with multicomponent reaction course. Afterwards, the various alcohols have been used as substrates for the P-MCR, the reaction yields were high in case of benzyl and cinnamyl alcohol derivatives, and low to moderate for alkyl alcohols. When allyl alcohol was applied, the formation of desired product was not observed.

Mironov et al. (2003) reported the Ugi reaction conducted in water. The authors have also shown that after the addition of cationic surfactant-cetylpyridinium chloride, the solubility of the substrates was increased, providing a series of β-lactams with higher yields than in pure water. The detailed studies on the Ugi reaction in the aqueous surfactant solution were also performed by Madej et al. (2017). Didodecyldimethylammonium bromide (DDAB) and Triton X-100 were determined to be the most suitable surfactants for the promotion of Ugi reaction. The presence of aggregates in these systems was confirmed by dynamic light scattering and fluorescence measurements in the presence of 1,1′-dioctadecyl-3,3,3′,3′-tetramethylindocarbocyanine perchlorate (DiI). Moreover, the detailed studies on the influence of the surfactants concentration and the reusability of studied system on the reaction course were performed. Finally, the positive effect of the addition of surfactant on the reaction yield has been proved delivering 26 different α-aminoacyl amide derivatives **9** with high yields.

## The kabachnik-fields reaction

**Scheme 6:**
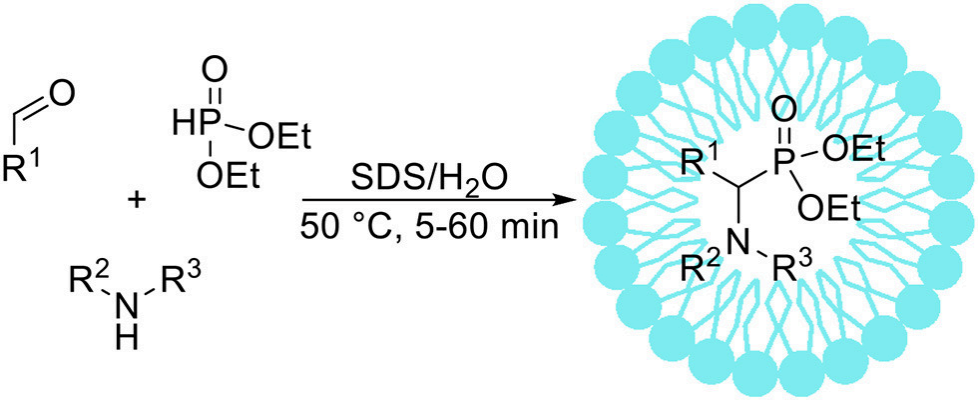
The Kabachnik-Fields reaction.

The Kabachnik-Fields reaction takes place between a carbonyl compound, an amine and hydrophosphoryl compound leading to α-aminophosphonates (Scheme 6). Lately, there is a considerable interest in Kabachnik-Fields synthesis of α-aminophosphonates, as they are structural analogs of the corresponding α-amino acids. In this context various biological activities of α-aminophosphonates are well documented (Atheron et al., 1986; Giannousis and Bartlett, 1987; Allen et al., 1989; Kafarski and Lejczak, 1991). Kabachnik-Fields reaction is usually catalyzed by Lewis acids, however there are issues connected with one-pot synthesis from a carbonyl compound, an amine and a phosphite ester due to decomposition or deactivation of the Lewis acid catalyst *via* water generated during the reaction course (Genet et al., 1992; Laschat and Kunz, 1992). Sobhani and Vafaee (2009) have reported the Kabachnik-Fields reaction conducted in an aqueous micellar SDS solution at 50°C. After short optimization studies the authors demonstrated the usability of micellar solution for efficient syntheses of 25 α-aminophosphonate derivatives. It is worth to mention that under optimized conditions the reaction was very fast-full conversion of substrates occurred after 5–60 min. Reddy et al. (2016) described the Kabachnik-Fields reaction conducted in Triton X-100 micellar solution. The authors reported short optimization studies, and investigated the antibacterial activity of 10 α-aminophosphonates synthetized with high yield.

## The strecker reaction

**Scheme 7:**
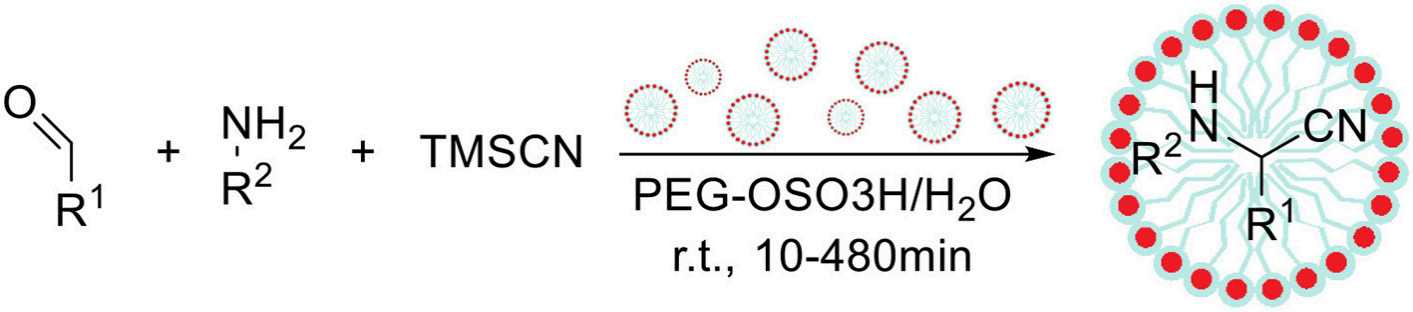
The Strecker reaction under micellar catalysis type 2 conditions.

The reaction described by Strecker between a carbonyl compound, amine and cyanide anion is one of the most powerful tools for the synthesis of α-aminonitriles, which can be further hydrolyzed to the corresponding α-amino acids (Scheme 7; Shafran et al., 1989). Among variety of catalytic systems for the Strecker reaction, Shekouhy (2012) reported the application of sulfuric acid-modified polyethylene glycol (PEG-OSO_3_H) as a Bronsted acid–surfactant combined catalyst (micellar catalysis type 2), which enabled to perform this reaction in water. The author has optimized the reaction conditions including solvent type, temperature and different catalytic systems based on sulfuric or Lewis acids. Under optimized conditions 42 α-aminonitriles were synthetized from various aldehydes or ketones, amines and trimethylsilyl cyanide with high yields (81–96%), proving usability of developed synthetic method. Finally, the studies on the reusability of catalyst showed that the catalytic system can be reused nine times without any loss in catalytic properties (the same product yield after 10 min). The extension of reaction time enabled to obtain the desired product even after 15 cycles with only slight drop in the yield.

## Kinugasa reaction

**Scheme 8:**
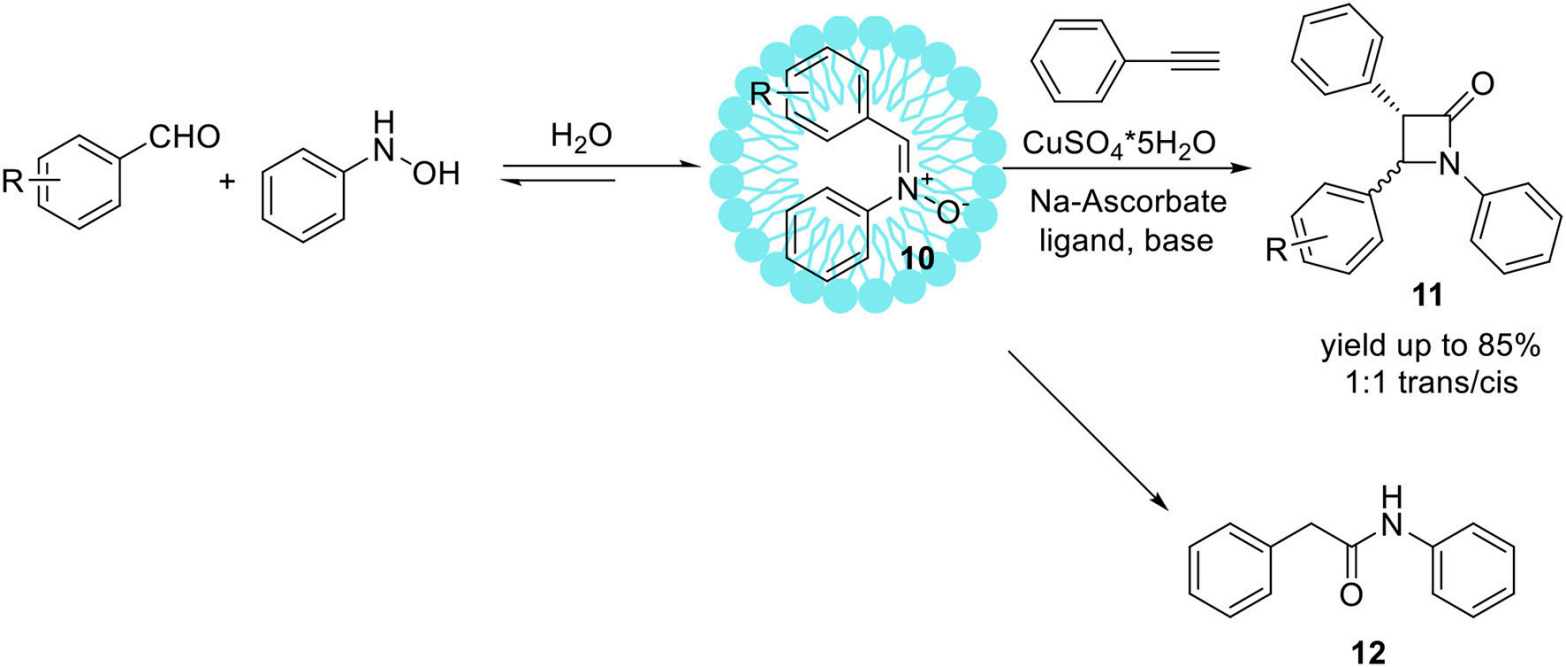
Micelles promoted synthesis of β-lactames *via* Kinugasa reaction.

The Kinugasa reaction is an efficient and convenient methodology for the obtaining of β-lactams from copper (I) acetylides and nitrones (Kinugasa and Hashimoto, 1972; Marco-Contelles, 2004). Usually, Kinugasa reaction is accomplished in organic media, such as MeCN, DMF or pyridine. Basak et al. have developed efficient Kinugasa reaction with copper catalyst, under aqueous conditions in the mixture of MeCN/water, DMF/water, and *t*-BuOH/water (Basak et al., 2007). However, the application of the pure water as a reaction media resulted in the decreasing of efficiency and yield. Comprehensive studies under various aqueous systems as the reaction medium for Kinugasa reaction furnishing high yields of β-lactams have been expanded. Based on previous results which show lower reactivity in pure water compared to aqueous-organic systems (Basak et al., 2007), micellar systems were engaged to replace the organic solvent improving the solubility of the substrates and the products, as well as promote the reaction by enhancing the concentration of the substrates inside the micelles. Chatterjee et al. published an elegant one-pot protocol toward the isoxazolines promoted by sodium dodecyl sulfate (SDS) or cetyl trimethylammonium bromide (CTAB) (Chatterjee et al., 2003). The emulsion droplets made of surfactants are sufficiently hydrophobic therefore protecting water-labile components e.g., nitrones from hydrolytic degradation (Manabe et al., 1999). Lorello and co-workers have recently applied this micellar system based on SDS for the synthesis of tyrosine analogous containing a cyclic α,β-unsaturated ketone ring which can be further modified by the reaction with C,N-diphenylnitrone in aqueous media (Lorello et al., 2008). In 2009, McKay et al. reported the synthesis of β-lactams **11** *via* multicomponent Kinugasa reaction in aqueous media promoted by SDS (McKay et al., 2009). Dehydrative nitrone creation from benzaldehyde and phenylhydroxylamine in the presence of SDS micelles proceeded in a short time resulted in C,N-diphenylnitrone **10** with 90% yield (Scheme 8).

The developed protocol provided β-lactams **11** with yields up to 85%. In addition to desired products **11**, an amide by-product **12** was also isolated. In general, an amide product is not a side-product of the discussed reaction and its formation may be exceptional for the engaged micellar-aqueous conditions. The high yields of hydrolytic resistant compounds provided under aqueous conditions features the reworked Kinugasa reaction conformable to metabolic labeling and bioorthogonal applications. The term bioorthogonal chemistry refers to chemical transformation that occurs inside of living cells without interfering with native biochemical processes (Prescher et al., 2004; Sletten and Bertozzi, 2009). The advances in Kinugasa reaction performed in micellar systems make this reaction useful for bioorthogonal tagging of cells by introducing nitrone or alkyne groups in their surfaces (Sherratt et al., 2014; Tra and Dube, 2014; Chigrinova et al., 2015). Reported developments in Kinugasa reaction conditions provided environmentally sustainable methods toward large scale synthesis of β-lactams in the absence of organic solvents.

## Hantzsch reaction

**Scheme 9:**
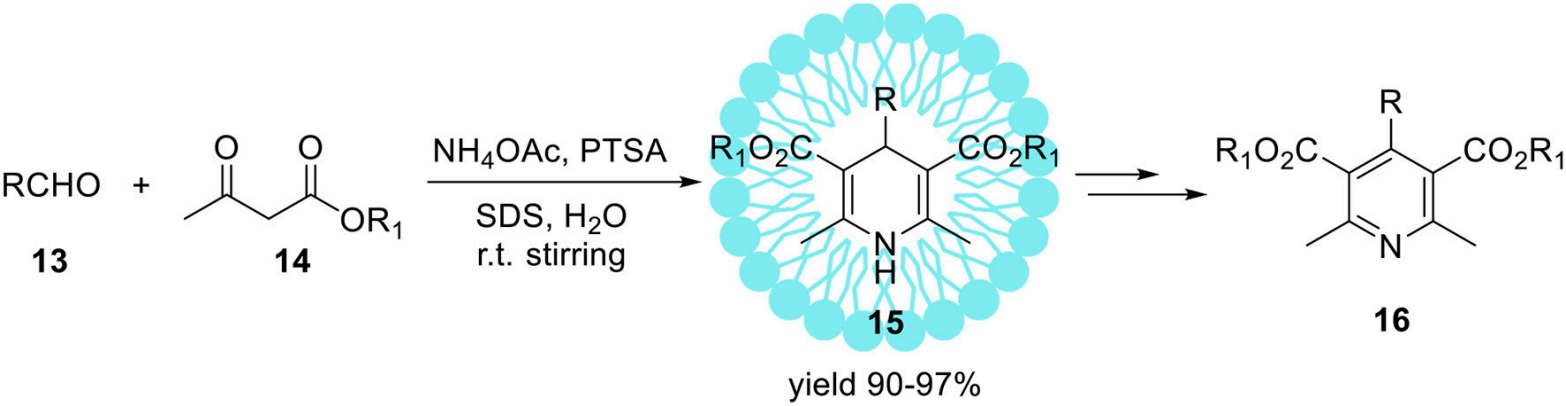
Micelles promoted synthesis of dihydropyridines **15** *via* Hantzsch reaction.

The Hantzsch reaction is a multi-component condensation between an aldehyde **13**, β-keto ester **14** and ammonium acetate or ammonia (Scheme 6). The early reaction product is a dihydropyridine **15** which can be subsequently oxidized to the corresponding pyridine derivative **16** (Scheme 6). The driving force for this second reaction step is aromatization. For the first time discussed reaction was reported in 1881 by Hantzsch (1881). Products of this reaction 1,4-dihydropyridines (1,4-DHP) are an important class of bioactive compounds in medicinal chemistry (Bossert et al., 1981). The standard method for the synthesis of 1, 4-dihydropyridines **15** is the one-pot reaction conducted in an acetic acid or by refluxing in alcohol (Hantzsch, 1888; Loev and Snader, 1965). This method suffers from disadvantages such as long reaction time, harsh reaction conditions, and low yields of products. Therefore, the refined synthetic protocols for the synthesis of 1,4-dihydropyridine derivatives are in continuous demand (Gordeev et al., 1996; Chari and Syamasundar, 2005). In 2008, Kumar et al. developed a practical protocol of the synthesis of Hantzsch ester derivatives **15** in aqueous-micellar system catalyzed by *p*-toluenesulfonic acid (PTSA) under ultrasonic irradiation (Scheme 9; Kumar and Maurya, 2008b).

**Scheme 10:**
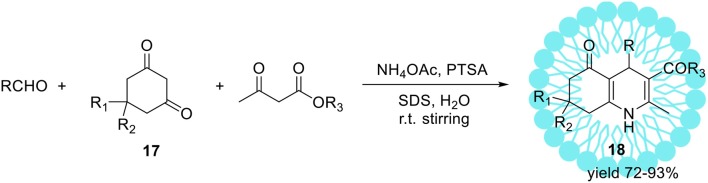
Micelles and ultrasonic promoted synthesis of polyhydroquinoline derivatives **18** *via* unsymmetrical Hantzsch reaction.

The same reaction performed in pure water (without SDS) resulted in product **15** with substantially lower yield 28%. Upon ultrasonic irradiation of the reaction mixture containing SDS, the final product **15** was isolated in 96% yield within 1 h. The application of organic solvents like methanol, ethanol, or THF as a replacement for water resulted in yield reduction. Finally, under optimized conditions series of 4-aryl-1,4-dihydropyridines were obtained with high yields up to 97%. Heterocyclic aldehydes such 4-pyridinecarboxaldehyde as well as aliphatic ones e.g. acetaldehyde were also found as suitable substrates resulted in 1,4-dihydropyridines with similar yields. The same authors reported the sufficient protocol for the synthesis of polyhydroquinolinederivatives **18** (Scheme 10) promoted by aqueous micelles under ultrasonic irradiation. To reach the goal they carried out the four-component condensation of an aldehyde, the cyclic 1,3-diketone **17**, an acetoacetatic ester, and ammonium acetate (Scheme 10) in the presence of PTSA as a catalyst.

Under optimized reaction conditions, several polyhydroquinoline analogous **18** were obtained with excellent yields up to 93%.

**Scheme 11:**
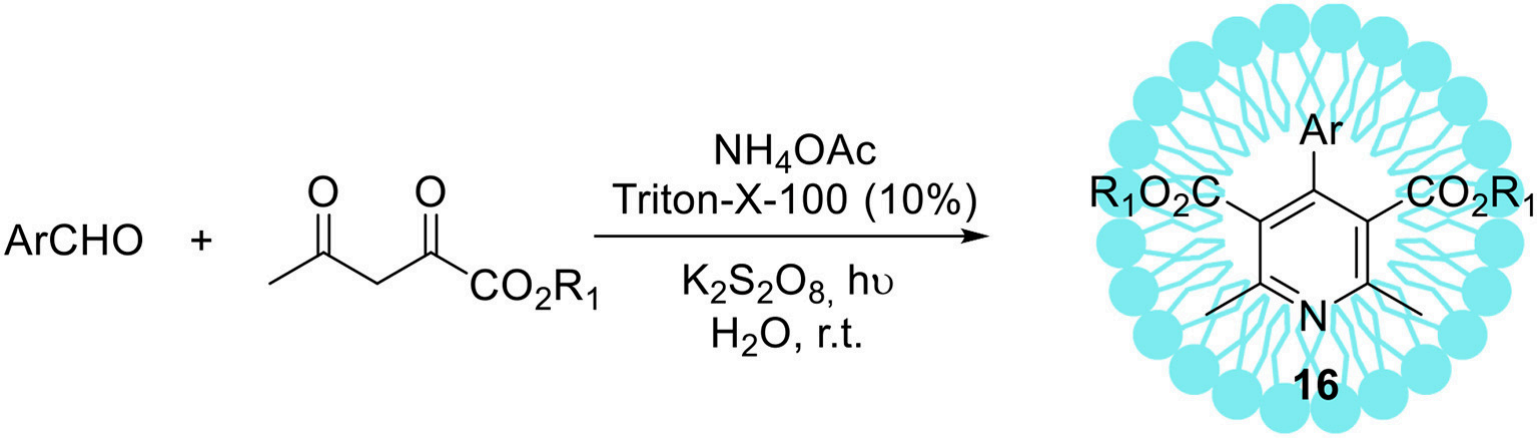
Micelles promoted synthesis of pyridine derivatives **16** *via* unsymmetrical Hantzsch reaction.

The non-ionic Triton X-100 is a commonly used surfactant which has found many submissions to biological systems as a solubilizer (Hinze and Pramauro, 1993; Zeng and Osseo-Asare, 2004). In addition to these applications, the synthesis of hexahydroquinoline derivatives **18** *via* a four-component Hantzsch condensation with various electronically and structurally divergent aldehydes promoted by aqueous-micellar system made of Triton X-100 has been recently reported (Scheme 10, R_1_ = R_2_ = Me) (Heravi et al., 2014). In contrast to the existing methods, the use of any potentially hazardous metal or Lewis acid catalysts is avoided. The same micellar-system was used for the synthesis of pyridine derivatives with excellent yields in a short time at ambient temperature, employing potassium persulphate in the presence of visible light (Scheme 11; Ghosh et al., 2013).

The recycling of the catalyst revealed that five consecutive runs did not affect substantially its catalytic activity (recovery amount 91% and yield 87%, after the 5th run). The operational simplicity in using aqueous-micellar systems as well as exceptional yields of the products obtained are the main benefits of this protocol. Furthermore, the application of environmentally sustainable Triton X-100 as a reaction promoter for the synthesis of pharmaceutically relevant compounds may be of great interest for industrial applications.

## Miscellaneous multicomponent reactions leading to formation of heterocyclic compounds

**Scheme 12:**
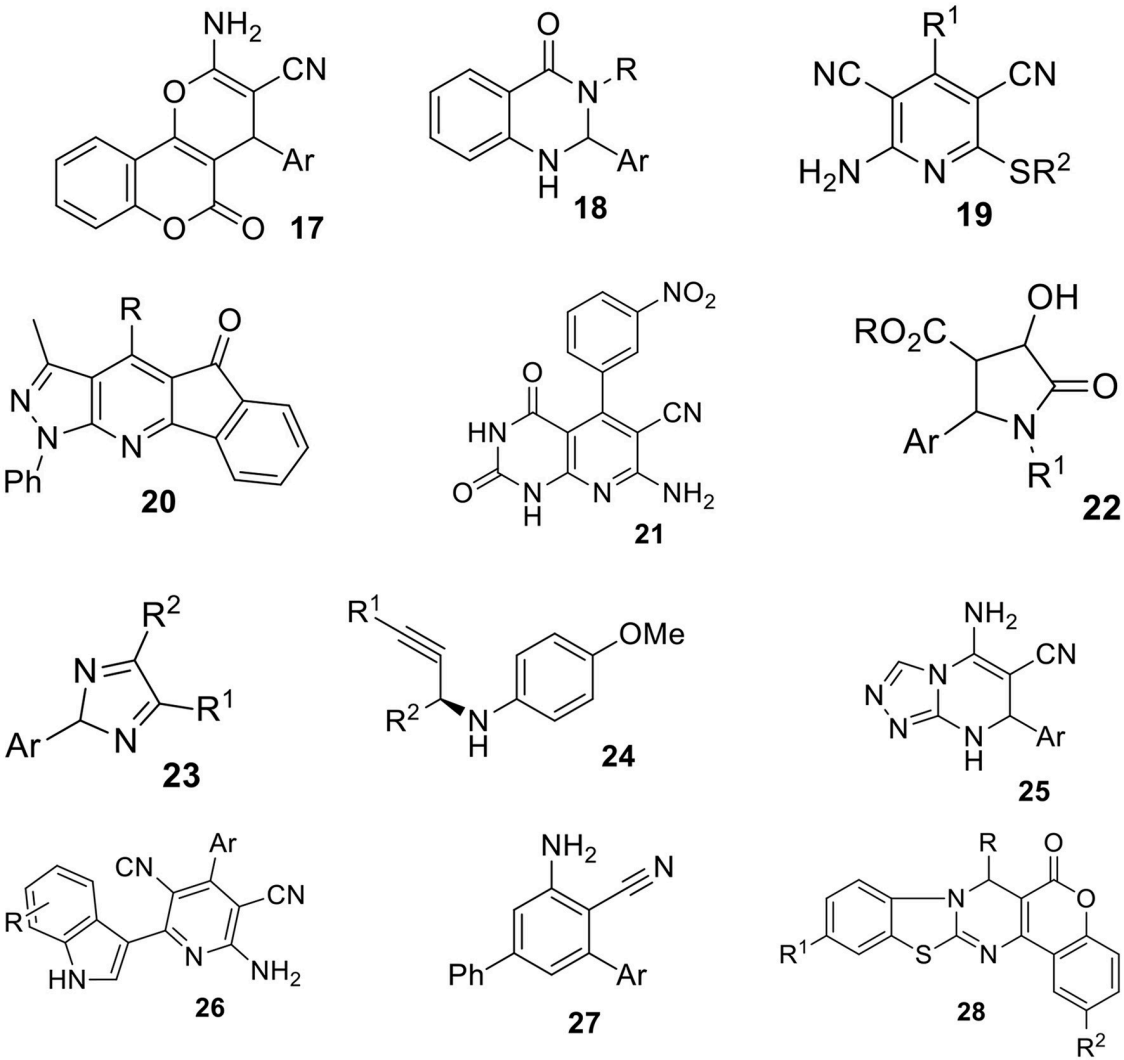
Structures of compounds **17**-**28** obtained via MCRs in aqueous micellar solutions.

**Scheme 13:**
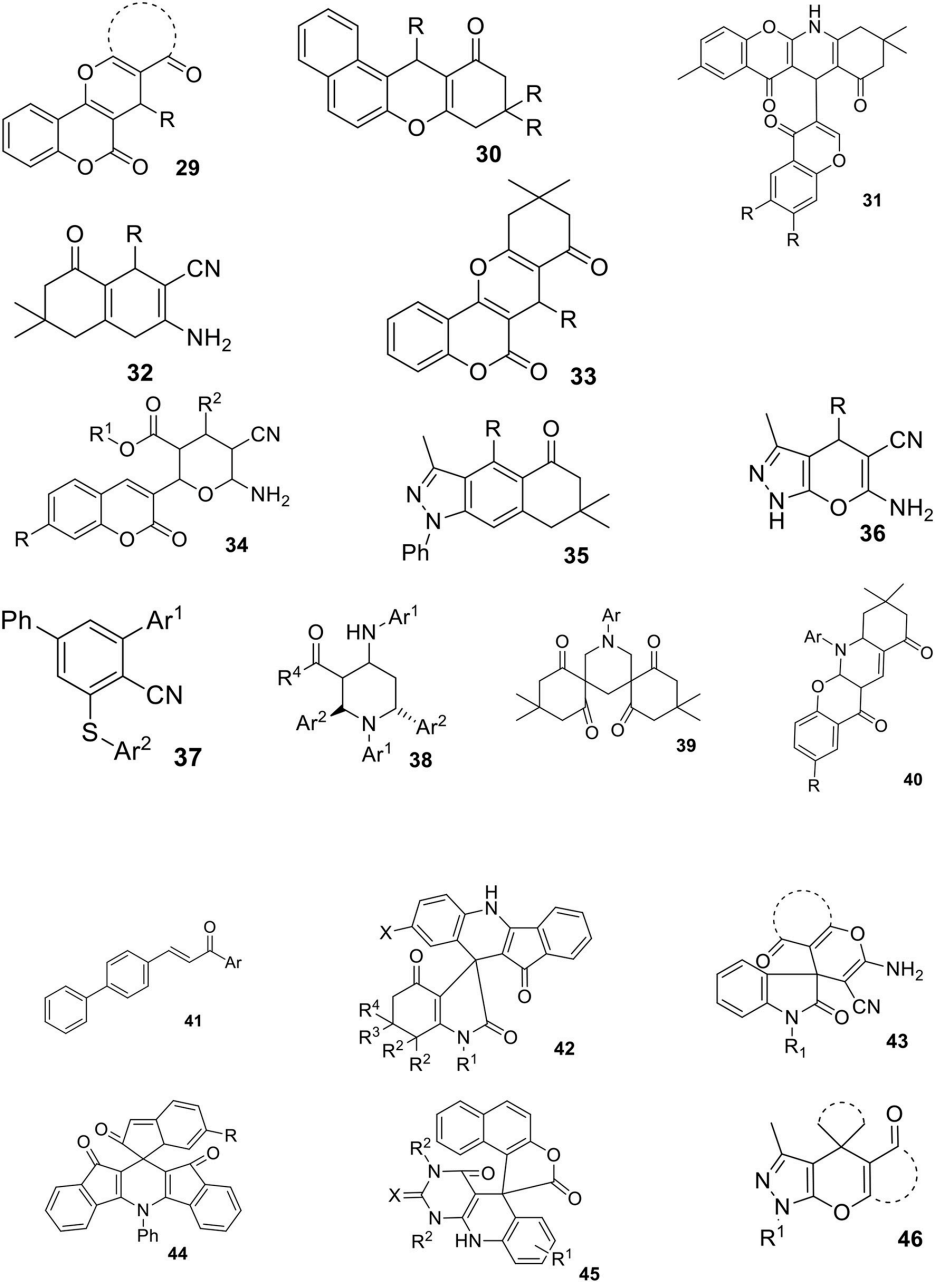
Structures of compounds **29**-**46** obtained via MCRs in aqueous micellar solutions.

**Scheme 14:**
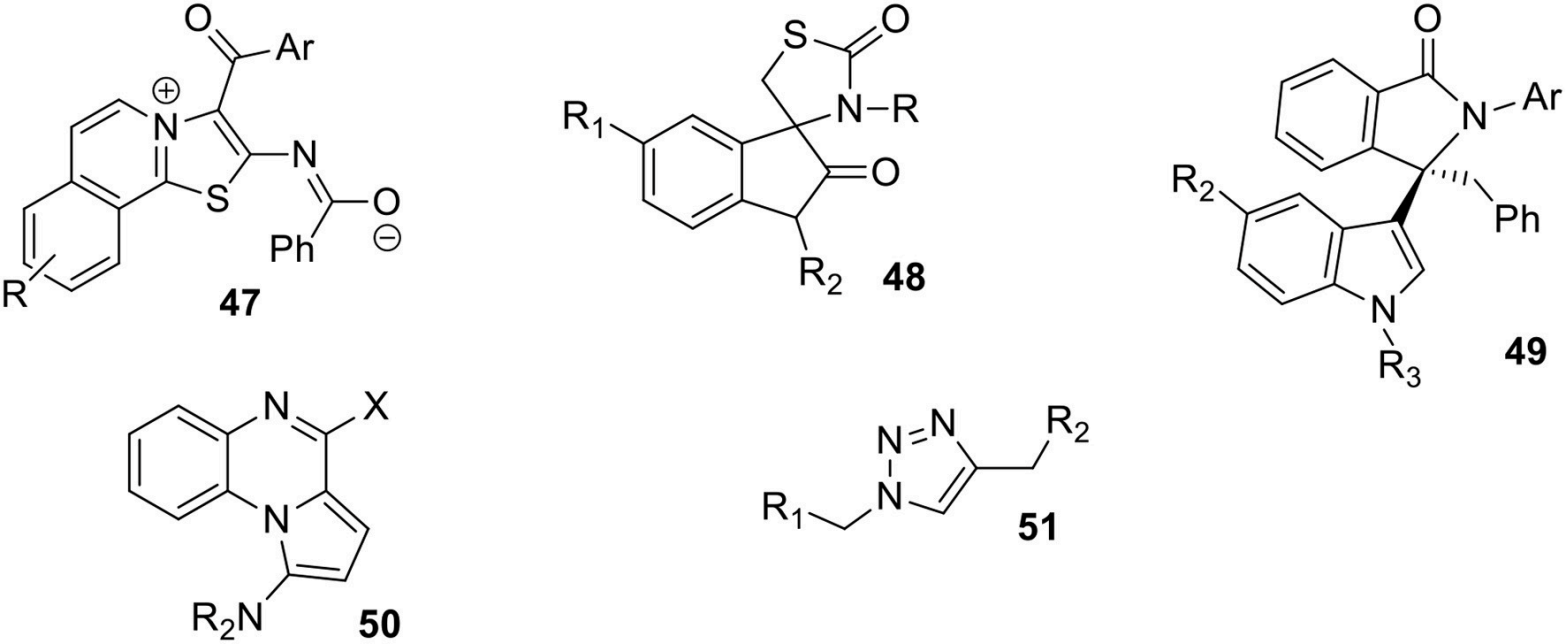
Structures of compounds **47**-**51** obtained via MCRs in aqueous micellar solutions.

The heterocyclic compounds are very important intermediates in organic chemistry, especially in medicinal chemistry due to their interesting biological activities. Therefore, the development of new multicomponent reactions can be recognized as attractive and efficient methods for synthetic organic chemists. In this part of review, we summarized different types of MCRs accelerated by aqueous micelles, which were recently described in the literature. In most cases, these reactions can be performed in water however, the addition of surfactant, leading to micelle formation, enhances the rate and yield of particular reactions. Some of the most relevant structures obtained *via* simple one-pot MCRs in an aqueous micellar media are shown on Schemes 12–14. It is important to note that attempted synthesis of those compounds in pure water failed what is a good evidence for the importance of micellar catalysis. All examples are summarized in Supplementary Table 1. This table provided product structures, reaction conditions, obtained yields and type of catalysis used.

Aryl substituted dihydropyrano[*c*]chromens (**17**) have a widespread biological properties such as plasmolytic, diuretic, anti-HIV, anti-anapylatic. Moreover, 4*H*-puran moieties can be useful as photoactive compounds as well. The multicomponent reaction from aryl aldehyde, malononitrile and 4-hydroxycoumarin or dimedone was performed in water in the presence of surfactant (Sheikhhosseini et al., 2013) (Supplementary Table 1, entry 1). The reaction conducted in pure water did not occur. The presence of DBSA micelles was necessary to efficiently carry out the reaction under micellar catalysis type 2 conditions. Under optimized conditions 8 derivatives of **17** were obtained with 69–90% yields. In Jafari and Ghadami (2016) verified other than DBSA aqueous surfactant medium (Supplementary Table 1, entry 1). The addition of surfactants like SDS, tetrabutylammonium bromide, and Triton X-100 proceeded with very low yield. However, the application of CTAB allowed to obtain product **17** with very high yield (85–99%).

Wang et al. (2008) reported the one-pot three-component reaction of aldehydes, primary amines and isatoic anhydride leading to the quinazolinone derivatives (**18**) in 77–86% yield (Supplementary Table 1, entry 2). Obtained products belong to an important class of compounds due to high biological activities reported as antibacterial, antifungal, anti-inflammatory, anti-HIV and anticancer. The use of Lewis acid-surfactant-combined (LASC) catalyst like [Zn(PFO)_2_] provided an efficient micellar system for the synthesis of **18**. LASC was recognized as both - a catalyst which triggers the substrates, and a surfactant which forms micelles or colloidal dispersion. The reaction did not occur without catalyst. To improve reaction rate, the authors used mixed solvents system (H_2_O:EtOH, 1:3, v:v) in the presence of LASC. It is important to note that the catalyst can be reused for at least three times without any change of activity and 15 different derivatives of **18** were obtained with good yields. In Sharma et al. (2012) verified other surfactants for the synthesis of **18** such as tetrabutylammonium bromide (TBAB), tetrabutylaminium hydrogen sulfate (TBAHS), tetramethylammonium iodine (TMAI) or SDS. The best result was obtained in the presence of aqueous solution of SDS. After detailed studies, the highest yield was obtained in mixed solvents system (H_2_O:EtOH, 3:1, v:v) in the presence of tartaric acid and SDS micelles.

The multicomponent reaction of aldehydes, malonitrile and *S*-alkylisothionium salts in the presence of SDS micelles yielding the pyridine-3,5-dicarbonitriles (**19**) was reported by Wang et al. (2009) (Supplementary Table 1, entry 3). All products were obtained within 60 min with very good yields (51–92%). The use of *S*-alkylisothionium salts instead of thiol ensures a lot of applications for the synthesis of complex molecules.

The synthesis of indeno[2′,1′:5,6]pyrido[2,3-d]pyrazole derivatives (**20**) from aromatic aldehyde, 1,3-indenedione and 5-amino-3-methyl-1-phenylpyrazole in the presence of SDS aqueous micelles was described by Shi et al. (2009) (Supplementary Table 1, entry 4). The series of 14 substituted aromatic aldehydes were tested leading to the desired products with excellent yields (90–98%).

The facile synthesis of pyridopyrimidine coumarin fused pyridine derivatives (**21**) in water was described by Bhattacharyya et al. (2013) (Supplementary Table 1, entry 5). The reaction between aminouracil/aminocoumarin, malonitrile and aldehydes in the presence of catalyst triethanolamine (TEAO) at 80°C in water gave desired products with very good yields (79–96%) for 23 pyridopyrimidines and coumarin fused pyridines. The reaction did not proceed without the addition of a catalyst. The TEOA acts as both-a surfactant and a Lewis base called as Lewis base-surfactant combined catalyst (LBSC), what indicates micellar catalysis type 2. The formation of vesicles was proved by dynamic light scattering (DLS), the aggregates about 100 nm diameter were observed. The optical microscopy finally confirmed the presence of vesicles in TEAO/water system.

The use of admicellar catalysis for the synthesis of polysubstituted pyrrolidinones (**22**) was reported by Sarkar and Murhopadhyay (2013) (Supplementary Table 1, entry 6). Admicelles are formed at lower surfactant concentration in water by the adsorption of surfactant on solid-liquid interface of nano-particels (Xu et al., 1996; Tyrode et al., 2008). The formation of admicelles has many practical applications such as flotation, petroleum recovery, food science, agriculture etc. (Zhu et al., 2012) and also can improve the efficiency of many reactions (Yuan et al., 2002; Marquez et al., 2007). The authors investigated the phenomenon of admicelles catalysis for the reaction between 4-methyl benzaldeyde, 4-chloro aniline and diethyl acetylenedicarboxylate ester at 30°C in aqueous medium in the presence of 0.8 mM CTAB (CMC value 0.92 mM). The TiO_2_ admicellar system allowed to synthesize **22** *via* three-component reaction with 70–83% yield. The TiO_2_/CTAB suspension was successfully reused up to five times with a slight decrease the yields (82–70%).

The use of PhIO for the micellar multicomponent reaction of pyrazole, aldehyde and phenyl hydrazine to afford pyrazole derivatives (**23**) was reported by Pal et al. (2014) (Supplementary Table 1, entry 7). Three various surfactants: CTAB (cationic), SDS (anionic), and Triton X-100 (neutral) were tested. The SDS micelles turned out to be the most suitable for the studied transformation. In the presence of PhIO/SDS aqueous system the desired products were obtained with higher yields than with other oxidants and Lewis acid catalysts. The formation of micelles increases the solubility of reactants and in next step the reactivity of PhIO. A wide range of substrates were investigated leading to respective **23** with very good yields (81–92%) at room temperature.

An enantioselective three-component reaction of aldehydes, amines, alkynes in the presence of bis(imidazoline)-Cu^I^ catalysts in the aqueous micellar medium was reported by Ohara et al. (2014) (Supplementary Table 1, entry 8). The reactions in the presence of SDS micelles gave propargylamines (**24**) with much higher yields than Triton X-100, CTAB or sodium laureate. In pure water only traces of product were obtained with moderate enantioselectivity. This methodology allowed to use a broad range of alkynes and aldehydes to obtain optically active **24** with excellent yields (up to 99%) and enantiomeric excesses (up to 99%).

Singh et al. (2014) reported the multicomponent synthesis of triazolopyrimidines (**25**) from aromatic aldehydes, malonitrile, and aminotriazole in the presence of boric acid in micellar media (Supplementary Table 1, entry 9. The influence of four surfactants on the reaction course was tested. The cationic surfactants CTAB was selected and gave **25** with excellent yields (80–96%) at 60°C within 20 min.

The synthesis of 4-aryl-2-aminocyanopyridine derivatives (**26**) containing indole moieties catalyzed by VB1 (thiamine hydrochloride) in micellar media was reported by Fatma et al. (2014) (Supplementary Table 1, entry 10). Among all tested surfactants, the most efficient for the four-component reaction was CTAB. When the reaction was carried out in the presence of CTAB, the **26** was obtained in shorter time with higher yield than with the other tested surfactants. Screening of different substrates resulted in products with excellent yields (90–92%). Moreover, the catalytic system was successfully reused.

The three-component reaction forming 4-aminopirimidine derivatives (**27**) from benzaldehyde derivatives, malonitriles and benzamidine hydrochlorides in micellar solution under reflux was reported by Aryan et al. (2015) (Supplementary Table 1, entry 11). The formation of desired product was not observed when the reaction was carried out without a surfactant. The effect of the addition of various surfactants on the reaction course was measured. Among the all investigated ones the most suitable was CTAB micelles. To validate the methodology the series of various aldehydes were used as substrates in the reaction, leading to **27** with very good yields (80–98%).

The synthesis of fused pyrimidines (**28**) in water catalyzed by a surfactant at room temperature was reported by Sahu (2016). The reaction between various aldehydes, 4-hydroxy coumarin, urea, 2-amino benzothiazole or 3-amino-1,2,4-traizole resulted in fused pyrimidines **28**. The most optimal surfactant for the discussed reaction was SLS (sodium lauryl sulfonate). Other tested surfactants like SDS or TBAB gave lower yields in comparison to SLS. Under optimized conditions 21 examined **28** were synthetized with high yields (Supplementary Table 1, entry 12).

The reaction between 4-hydroxycoumarin, aldehydes, and 1,3-diketocompounds (like dimedone, cyclohexane-1,3-dione, or ethyl acetoacetate) described by Pradhan et al. (2013) gave chromeno[4,3-b]chromene derivatives (**29**) (Supplementary Table 1, entry 13). This transformation was also efficiently promoted and catalyzed by a LASC, [Fe(DS)_3_] mixture in water at 70°C. This catalytic system tolerates harsh reaction conditions such as high temperature. After detailed optimization studies, including various LASCs derivatives, 28 derivatives of **29** were obtained with 70–93% yield. Additional DLS studies have shown that [Fe(DS)_3_] creates in aqueous solution colloidal aggregates with around 140 nm in diameter. This micellar system can be reused up to four times without substantial loss of activity.

Mirjalili et al. (2012) reported the multicomponent reaction of tetrahydrobenzo[*a*]xanthenes-11-one derivatives (**30**), aldehyde, dimedone and β-naphtol in micellar media (Supplementary Table 1, entry 14). The best results were obtained using Bi(NO_3_)_3_ · 5H_2_O as catalyst and SDS micelles. The formation of product in pure water was not observed. To validate the methodology the series of various aldehydes and diketones were used as substrates to obtain **30** with very good yields (74–86%).

Micellar system can be also used for the selection of particular products formation. In the micelle promoted multicomponent reaction reported by Ghosh et al. (2015) (Supplementary Table 1, entry 15) one product containing chromeno[2,3-b]quinoline skeleton (**31**) was formed with good yield. The one-pot three-component reaction carried out between 2-aminochromone, chromone-3-carbaldehyde, and dimedone in an aqueous micellar medium leaded to the formation of target products **31** with very good to excellent yields. Additional studies on the influence of various reaction condition including solvents and aqueous solution of surfactants were performed. The most efficient reaction medium turned out to be aqueous SDS solution at 80°C. Again, the product formation was not observed in pure water. The use of micellar system allowed to obtain derivatives of **31** in higher yields in respect to common solvents such as methanol or DMF.

Sheikhhosseini et al. (2013) reported the multicomponent reaction of aldehyde, malonitrile, dimedone in water in the presence of DBSA micelles (Supplementary Table 1, entries 16). The reaction in pure water did not occur. Under detailed studies 8 dihydropyrano[c]chromenes (**32**) were obtained with 71-90% yields.

In several cases the aqueous micellar system was found to be great medium to improve selectivity of particular reactions. Ganguly et al. (2014) performed studies on the synthesis of 7-arylbenzopyrano[4,3-b]benzopyran-6,8-dione derivatives (**33**). The reaction proceed in water resulted in the formation of two bi-component products *via* condensation of benzaldehyde with 4-hydroxycoumarin or dimedone with 10 and 22% yield, respectively. The addition of anionic surfactant SDS into water improved the selectivity of mentioned reaction provided **33** with 76–94% yields (Supplementary Table 1, entry 17).

The synthesis of fluorescent coumarin-4*H*-pyran derivatives (**34**) *via* three-component reaction of different β-ketoesters, benzaldehyde and malonitrile in micellar media at 80°C was reported by Omar et al. (2017) (Supplementary Table 1, entry 18). The desired product was obtained efficiently in aqueous ethanol (EtOH:water; 1:1; v:v) in the presence of cationic surfactant cetyltrimethylamonium chloride (CTAC). 13 derivatives of **34** were synthetized under optimized conditions with yields of 30–85%.

Another example of synthesis of fused pyrazoles was reported by Wang and Shi (2012), which described the obtaining of pyrazolo[3,4-b]quinolone-5(6*H*)-one derivatives (**35**) in the presence of SDS micelles as reaction medium at 90°C (Supplementary Table 1, entry 19). This three-component reaction of aromatic aldehyde, 5,5-dimethyl-1,3-cyclohexanedione and 3-methyl-1-phenyl-1H-pyrazol-5-amine was performed with excellent yields (84–98%). Additionally, the catalytic system was reused 5 times without significant change in activity.

The four-component reaction for the synthesis of dihydropyrano[2,3-c]pyrazoles (**36**) in water with the presence of cocamidorpopropyl betaine (CAPB) was reported by Tamaddon and Alizadeh (2014). Various surfactants such as CTAB, SDS, Triton X-100, Triton X-114, PEG- 400, and CAPB were tested giving the respective **36** with yields 82–98%. The best results was obtained with the addition of the zwitterionic surfactant CAPB resulting in the highest reaction efficiency. The main advantages of using CAPB is that the formation of side products was not observed and short reaction time at 50–60°C. Moreover, the reaction medium can be recycled (Supplementary Table 1, entry 20).

The four-component reaction of acetophenone, arylaldehydes, arylthiol and malonitryle in the presence of Triton X-100 micelles providing polisubtytuted piridines (**37**) was reported by Heravi and Soufi (2015). The using of triton X-100 micelles system gave product with much higher yield than in another reaction medium. The corresponding **37** were obtained with high yields (80–95%) for 21 piridines (Supplementary Table 1, entry 21).

An interesting example of multicomponent reaction proceeded “on water” was proposed by Parikh et al. (2016) concerning the synthesis of highly substituted tetrahydropyridines (**38**) from aldehydes, amines and 1,3-dicarbonyl compounds in 2:2:1 molar ratio (Supplementary Table 1, entry 22). The efficiency of the reaction carried out without any catalyst in water was low. Among all tested surfactants, only anionic surfactants such as SDS and SDOSS (dioctyl sulfosuccinate) gave satisfactory results. The use of organic solvents in the presence of catalytic amount of SDOSS resulted in lower yields and emphasized the particular role of water by the formation of microreactors on the water surfactant interface. The presented five-component reaction *via* a tandem inter- and intramolecular Mannich reaction allowed to obtain 17 derivatives of **38** with good yields (63–86%) and high diastereosectivity.

3,5-dispirosubsstituted piperidines (**39**) possesses antibacterial activity comparably to standard drugs. Lohar et al. (2016) presented the reaction between amines, a formaldehyde and a dimedone using iron (III) trifluoroacetate Lewis acid in the presence of SDS micelles at room temperature (Supplementary Table 1, entry 23). The derivatives of **39** were obtained with very good yields (75–88%). Besides, the catalytic system was used five times with only a slight decrease of yields.

The multicomponent synthesis of chromeno[2,3-b]quinolones (**40**) *via* reaction between an amine, a chrome-3-aldehyde and dimedone in micellar medium with high yields (80–98%) was reported by Ghosh et al. (2014). The reaction carried out in water in the presence of TBAB (Supplementary Table 1, entry 24). The authors tested active methylene components such as dimedone, Meldrum acid, indane-1,3-dione, 4-hydroxycoumarin and acetyloacetate. However, only dimedione was accepted in the three-component reaction. The scope of substrate was verified with respect to the amine and presented results showed a superiority of reaction in micellar media than in acetonitrile. Moreover, the reusability of the TBAB/H_2_O system was investigated and the filtrate was reused in seven cycles.

The multicomponent cascade reactions of biaryl-based chalcones (**41**) in pure water and micelles were reported by Armenise et al. (2016). The presented Pd-catalyzed, ligand-free aerobic Suzuki-Miyaura reaction in water provided complex biarylchalcones with excellent yields for some **41**. In this case the use of lipophilic ketones was necessary to improve reaction yield by applying additive, because yields obtained in water were about 10%. Among all tested additives such as TBAB, SDS, Triton X-100, and C18-OPC, the newly obtained surfactant C18-OPC was the most effective. The use of aqueous micellar media was crucial to obtain more complex of **41** from substrates involving poorly reactive ketones. Moreover, a catalytic system was recycled without substantial loss of activity (Supplementary Table 1, entry 25).

Mondal reported the reaction between enaminones of dimedone, indane-1,3-dione and isatin leading to highly substituted spiro[indolo-3,10′-indeno[1,2-b]quinolin]-2,4,11′-trionesin (**42**). The reaction conducted in water containing cetyltrimethylammonium bromide (Supplementary Table 1, entry 26). The other solvents were also examined however the yields of obtained products were much lower in respect to water-CTAB micellar system. This methodology was applied for the synthesis of 75 derivatives of **42** with excellent, over 91% yields (Mondal et al., 2014).

The heterocyclic moiety based on spirooxindole derivatives (**43**) is a core structure in various pharmaceuticals and natural products e.g., cytostatic alkaloids such as spirotryptostatins A, B and strychnophyline (Cui et al., 1996). The reaction between isatin, malonitrile and 1,3-dicarbonyl compounds proceeded in water with 30% yield. To improve reaction efficiency, Wang et al. (2010) reported the protocol in micellar system which promoted multicomponent reaction providing target spirooxindols with fused chromones. Careful optimization of reaction conditions, including temperature and various additives was necessary. The use of sodium stearate, anionic surfactant solution allowed to increase the reaction yield from 30 to over 91%, After detailed optimization, 16 derivatives of **43** were synthetized with high yields exceeding 89% (Supplementary Table 1, entry 27).

Spiro[diindenopyridine-indoline]trione derivatives (**44**) are important class of compounds which exhibit strong fluorescence, with large Stokes shift what is of great interest in biochemistry and material sciences. Commonly, these compounds were obtained in the presence of acetic acid or sulphuric acid what hampered purification procedures. The reaction between 1,3-indandione, aromatic amines and isatins can be performed in water containing micelles of PEG-OSO_3_H what enhances reaction rate and yield (Sidhu et al., 2015). The synthesis of various **44** in PEG-OSO_3_H micellar solution proceeded in 10 min with good yields (88–97%). The catalyst was reused up to 4 times without significant loss of its catalytic activity. The same reaction can be performed under solvent free conditions using PEG-OSO_3_H alone (Supplementary Table 1, entry 28). However, in this case the separation of product was difficult what diminished the yield of reaction.

Spiro[dihydroquinoline-naphthofuranone] derivatives (**45**) exhibit potential biological and pharmacological activities. After investigating the effect of solvents on the reaction rate, water was chosen as the most sui‘ medium. The main factor promoting the synthesis of **45** from isatins in micellar system (Kong et al., 2017) is considered the hydrogen bonding effect (Supplementary Table 1, entry 29). It is justify by the fact that the formation of hydrogen bonds between water and substrates resulted in the more polar transition states than initial ones (Butlerm and Coyne, 2010). Moreover, the use of SDS micelles resulted in much higher yield than results obtained in pure water. To verify the effect of water on the reaction course, the model reaction was performed under two different conditions: 10 wt.% SDS/H_2_O and 10 wt.% SDS/D_2_O. The product with higher yield was obtained in the presence of H_2_O. Both, H_2_O and D_2_O have similar hydrophobic and polarity effects, so hydrogen-bonding effects are the main factors, which influence the efficiency of reaction studied. The reactions of isatins, 2-naphthol and barbituric acids were performed for 24 derivatives of **45** with very good yields (80–92%). The medium was reused at least 5 times without significant decrease in the reaction yield.

Tricyclic 4-spiro pyrano[2,3-c]pyrazoles (**46**) possess a potent bioactivity as inhibitors of acetylohinesterase (Sunazuka et al., 2002), γ-secretase (Wu et al., 2013), an anti-angiogenic activity against human umbilical vein endothelial cells (Hayashi et al., 2009). Mukherjee et al. (2015) reported the DBSA, Brönsted acid-combined catalyst that efficiently catalyzed the reaction between pyrazolone, cyclic 1,3-dicarbonyls and cyclic ketones providing **46**. This reaction did not occur in pure water or an organic solvent, however under micellar catalysis type 2 conditions various **46** were obtained in good yields (75–96%) at 90°C (Supplementary Table 1, entry 30).

An another example of multicomponent reaction is the synthesis of thiazo[2,3-α]isoquinolin-4-ium (**47**) proposed by Maity et al. (2014) (Supplementary Table 1, entry 31). The protocol described a new group of mesonic compounds obtained from isoquinolines, 2-bromoacetophenones and benzoyl isothiocyanate in micelles promoted reaction. Four different surfactants were tested. The mixture of Amberlite IRA 402(OH) and cationic surfactant CTAB was the most suitable for reaction and led to desired products with the highest yields. It is important to note that the reaction did not occur without surfactant. Under optimized conditions 18 derivatives of **47** were synthetized with yields of 80–91%. The reaction medium can be recycled up to 5 times with only slight decrease in the yield (91–67%).

The synthesis of spiro[indoline-3,2′-thiazolidinone] derivatives **48**, pharmaceutically important compounds, *via* multicomponent reaction was recently proposed by Preetam and Nath (2016) (Supplementary Table 1, entry 32). The authors reported a one-step synthesis of **48** from primary amines, isatin derivatives and thioglycolic acid in the presence of DBSA micelles at room temperature with 76–87% yields. DBSA is an efficient Brönsted acid surfactant combined catalyst, what indicates micellar catalysis type 2.

The multicomponent reaction of 2-indo-*N*-phenylbenzamides, terminal alkyne and substituted indoles in aqueous micellar medium providing indolylisooidolinone derivatives (**49**) was described by Sarkar et al. (2013) (Supplementary Table 1, entry 33). Previously, the indolylisooidolinones were obtained in high-boiling, polar solvents like DMF what created the purification issue. However, the use of micellar catalysis can overcome this limitation. Detailed optimization studies were performed including various copper salts, ligands, surfactants and bases. The highest reaction yield was observed when polyoxyethanyl-α-tocopheryl sebacate (PTS) was used as a surfactant at 80°C. To validate the methodology different *N*-substituted benzamides and indoles were tested what allow to obtain desired products **49** with 32–80% yields. The presented catalytic system is less expensive than the heavy metal based catalysts for this kind of reaction.

The synthesis of 1,4-disubstituted pyrrolo[1,2-a]quinoxalines (**50**) by the multicomponent reaction of 3-substituted-2-chloroquinoxalines, propargyl alcohol, and secondary amines, catalyzed by Pd/Cu, in the presence of SDS micelles was proposed by Keivanloo et al. (2016). The reaction yield was decreased in the absence of a surfactant or in organic solvents. The addition of SDS significantly improved the course of reaction. The use of micelles is crucial for the success of the cross-coupling reaction. The derivatives of **50** were obtained under inert atmosphere at 80°C with 65–87% yields (Supplementary Table 1, entry 34).

The use of micellar system improved selectivity of multicomponent reaction was also reported by Tasca et al. (2015). The authors showed two steps synthesis of 1,2,3-triazoles (**51**) including: the *in situ* formation of the organic azide and click reaction through organic azide and alkyne. The reaction between organic bromides, sodium azide and alkyne to triazole catalyzed by 1 mol% of [Cu-(IMes)Cl] in water was promoted by the addition of SLS or TPG-750-M at room temperature within few hours. In order to validate the catalytic system 21 triazoles were obtained in three different variants: in pure water, in the presence of TPGS-750-M and SLS. In most cases the use of SLS was more efficient and also improved the regioselectivity. The reactions conducted in the presence of micelles allowed to obtain the **51** with very high yield (up to 98%). However, when reaction was performed in the presence SDS, CTAB or Triton X-100, the main product was an azide. These results definitely focused on great impact of micelles on reaction rate (Supplementary Table 1, entry 35).

## Conclusions

In this review, we have highlighted the usefulness of aqueous-micellar systems in multicomponent reactions. Moreover, to clarify the impact of the used surfactants on the reaction course we have organized and classified the reviewed MCRs in to two types; first-promoted by increasing solubilization of substrates by hydrophobic micellar medium and second-additionally catalyzed by active functional groups being an integral part of used surfactant. The classification is sometimes not straightforward, compared to classical approach in organic solvents or pure water. Compared with conventional protocols, the application of aqueous-micellar systems offers several advantages. Reactions become environmentally friendly by exclusion of toxic organic solvents. In most cases for reaction completion shorter reaction times is required and products were obtained in higher yields. Also the safer operation conditions, simple work up procedures, and efficient recovery and recycling of catalysts were noted. Furthermore, aqueous-micellar systems offer an unique hydrophobic environment providing sufficient protecting shield for water unstable reagents e.g., metal catalysts, isocyanides. Additionally, the reaction performed under micellar conditions often characterizes by high chemoselectivity, which is unreachable under standard conditions, what made it a superior approach for the preparation of highly complex particular heterocyclic molecules of medicinal concern. The wide availability of surfactants and their generally low cost are definite benefits for the laboratory syntheses. As a result, these processes show that greener alternatives may be used in respect to conventional approaches for the synthesis of synthetically relevant compounds. Last but not least, aqueous-micelles systems with catalytic groups may catalyze completely new types of reactions, providing products with intriguing properties.

## Author contributions

AM organized the database for Supplementary Table 1. DP, AM, DK, AB, and RO wrote sections of the manuscript. All authors contributed to manuscript revision, read and approved the submitted version.

### Conflict of interest statement

The authors declare that the research was conducted in the absence of any commercial or financial relationships that could be construed as a potential conflict of interest.
